# Adaptive soft sensor using stacking approximate kernel based BLS for batch processes

**DOI:** 10.1038/s41598-024-63597-5

**Published:** 2024-06-04

**Authors:** Jinlong Zhao, Mingyi Yang, Zhigang Xu, Junyi Wang, Xiao Yang, Xinguang Wu

**Affiliations:** 1 Chinese Academy of Sciences, Shenyang Institute of Automation, Shenyang, China; 2 University of Chinese Academy of Sciences, Beijing, China; 3 Institutes for Robotics and Intelligent Manufacturing, Chinese Academy of Sciences, Shenyang, China; 4 Xi’an North Huian Chemical Industries Co., Ltd, Xi’an, China

**Keywords:** Batch process, Ensemble framework, Broad learning system (BLS), Kernel learning, Adaptive soft sensor, Chemical engineering, Computer science, Software

## Abstract

To deal with the highly nonlinear and time-varying characteristics of Batch Process, a model named adaptive stacking approximate kernel based broad learning system is proposed in this paper. This model innovatively introduces the approximate kernel based broad learning system (AKBLS) algorithm and the Adaptive Stacking framework, giving it strong nonlinear fitting ability, excellent generalization ability, and adaptive ability. The Broad Learning System (BLS) is known for its shorter training time for effective nonlinear processing, but the uncertainty brought by its double random mapping results in poor resistance to noisy data and unpredictable impact on performance. To address this issue, this paper proposes an AKBLS algorithm that reduces uncertainty, eliminates redundant features, and improves prediction accuracy by projecting feature nodes into the kernel space. It also significantly reduces the computation time of the kernel matrix by searching for approximate kernels to enhance its ability in industrial online applications. Extensive comparative experiments on various public datasets of different sizes validate this. The Adaptive Stacking framework utilizes the Stacking ensemble learning method, which integrates predictions from multiple AKBLS models using a meta-learner to improve generalization. Additionally, by employing the moving window method—where a fixed-length window slides through the database over time—the model gains adaptive ability, allowing it to better respond to gradual changes in industrial Batch Process. Experiments on a substantial dataset of penicillin simulations demonstrate that the proposed model significantly improves predictive accuracy compared to other common algorithms.

## Introduction

Batch process, essential in specialized chemical and pharmaceutical production, faces challenges such as high nonlinearity, significant variations between batches, and difficulties in real-time measurements^1^. As intelligent manufacturing advances, there’s a pressing need for enhanced control and management beyond traditional DCS systems^2^ to achieve integrated production goals. Although sensors implemented in the form of physical entities are widely used in industrial practice, the data they collect is not fully utilized and may eventually evolve into data garbage^3^. Installing numerous sensors is impractical due to costs associated with calibration and maintenance. Therefore, as an alternative to physical sensors, soft sensors has been increasingly popular in both academia and industry in recent years^4–8^.

Soft measurement technology involves processing measurement data and is divided into two types: model-driven and data-driven. Model-driven methods use mathematical models and require deep knowledge of chemical and physical processes, which can be difficult in complex industrial settings. Data-driven approaches, on the other hand, rely on historical data for modeling and prediction, reducing the need for specific process knowledge^9^. They identify patterns in large datasets commonly found in industrial environments, enabling accurate soft sensing models that provide effective estimation and control. Data-driven soft measurement models use statistical and machine learning methods to predict or estimate key system indicators by analyzing large datasets. Popular linear models such as principal component regression (PCR)^10^, partial least squares (PLS)^11^, and independent component regression (ICR)^12^ are valued for their statistical basis, interpretability, and ability to manage data collinearity. However, they struggle with highly nonlinear industrial processes. To address this, researchers are turning to soft sensing models based on support vector machines (SVM)^13,14^ and artificial neural networks^15–17^, which have strong nonlinear modeling capabilities. Especially the soft sensing models based on artificial neural networks, are gaining attention for their effectiveness in handling complex nonlinear industrial processes, providing robust tools for accurate estimation and prediction of key system parameters.

The application of deep learning-based soft sensing models in industry is widespread due to their ability to handle complex feature representations effectively. Deep learning architectures excel at capturing and learning nonlinear relationships in input data, particularly in highly nonlinear industrial scenarios^18–20^. However, these methods are computationally demanding and highly sensitive to hyperparameters, especially with large-scale data and complex network structures. Training and optimization can be time-consuming and costly, complicating their application to batch process, which often require rapid adaptation. To address these challenges, a new approach known as broad learning system (BLS) has been introduced^21^. Unlike deep learning, BLS offers a simpler structure, achieved by expanding the width of a single-layer neural network. This simplicity results in faster training speed and lower computational complexity. BLS’s flexible expansion capability allows it to adapt to new feature nodes and enhanced nodes without requiring retraining, enabling real-time adjustments. These characteristics make BLS an effective solution for handling highly nonlinear industrial process data while mitigating the complexities and time-consuming aspects of deep learning. Moreover, BLS has been mathematically proven to possess general approximation properties^22^ and has demonstrated fast training speed and accuracy comparable to deep neural networks on standard datasets like MNIST^23^ and NORB^24^. Thus, BLS offers a more reasonable and efficient modeling option for industrial applications.

With the continuous development of BLS, more and more variants of BLS are being applied in industrial applications. Fei Chu proposed a weighted broad learning system (WBLS) based on BLS to address noise and outlier problems in industrial processes^25^. A batch process fault detection method for multi-stage broad learning system was proposed by Chang Peng^26^. Miao Mou proposed a gated broad learning system based on deep cascaded for soft sensor modeling of industrial process^27^. Wenkai Hu introduces a novel fault diagnosis method utilizing a weighted timeliness broad learning system (BLS) with multi-feature extraction, enhancing process safety in modern industrial facilities by effectively addressing complex signal variations and interrelated faults^28^. Zhang et al. proposed a modified BLS (broad learning system) algorithm called CEBLS-dense, which uses a cascade of enhancement nodes with dense connections to modularize the enhancement nodes, effectively reducing the risk of redundant information and overfitting^29^. Jin et al. proposed manifold-based binary label smoothing (BLS) models with flexible labels, emphasizing soft label movement and adaptive target matrix learning, optimized using efficient iterative algorithms, which can be more beneficial for object and scene recognition problems^30^. Men et al. propose a novel Adaptive imbalance modified online broad learning system (AIM-OBLS) to improve fault diagnosis in imbalanced chemical process data streams, using niche techniques, oversampling, and manifold regularization to boost accuracy and efficiency^31^. However, these methods and standard BLS still faces significant difficulties in processing high-dimensional data because the features learned from shallow structures are not representative enough. The formal analysis of these shallow structural defects can be found in^32^, which indicates that shallow structures are far less expressive than deep structures. Therefore, in order to improve the complexity of representation, a more complex structure that enhances the cascaded connections between nodes and feature nodes has been proposed, and some variants have been developed based on this, including fuzzy BLS^33^, recurrent BLS^34^, etc. Among them, Yu proposed a progressive ensemble kernel-based BLS (KBLS)^35^.Specifically, it is to replace the enhanced nodes in BLS with feature projections learned by kernel functions. Mapping data to a higher dimensional space through kernel functions can transform data that was originally linearly inseparable in a lower dimensional space into data that is linearly separable in a higher dimensional space. This greatly enhances the nonlinear processing ability of BLS, while effectively reducing the uncertainty caused by random mapping, making the data more distinguishable and having a fixed number of hidden nodes, improving model performance. However, it should be noted that the introduction of kernel functions leads to significant computational efficiency, especially in the case of large-scale data. The complexity of these features hinders the reconstruction process required for Batch Process of dynamic characteristics, making it difficult for KBLS to quickly adapt to frequent changes.

In order to accelerate the training of kernel machines, Ali Rahimi proposed a method called random fourier features^36^. This method maps the input data to a randomized low-dimensional feature space and then apply existing fast linear methods. The features are designed so that the inner products of the transformed data are approximately equal to those in the feature space of a user specified shift-invariant kernel, thereby accelerating the training of the kernel machine. Nowadays, more and more researchers are using the method of random Fourier features to approximate kernel matrices, which not only reduces computational burden but also provides competitive performance^37–39^. Therefore, this paper aims to address the issue of time-consuming establishment of KBLS models and will use random Fourier features to establish the approximate kernel based BLS models(AKBLS). This strategy can not only significantly improve the nonlinear processing ability of the BLS model, but also minimize the time required for model establishment. A Stacking AKBLS model based on Stacking ensemble learning has been designed to enhance the model’s generalization ability, compared to simply integrating the model together through voting combinations. Finally, in order to better cope with the time-varying characteristics of Batch Process, this paper adopts the moving window method to establish the Moving window-Stacking AKBLS(MW-Stacking AKBLS) model, introducing certain adaptive capabilities to the model. The MW method updates the dataset window by merging newly measured samples and discarding old ones. Usually, the selected dataset is considered the most relevant to the current process. Every time the window is updated, the latest information about the process is obtained, so the constructed model can effectively describe the current state even as the process gradually changes^40^. The purpose of these methods aims to comprehensively improve the performance and adaptability of soft sensing models, and better apply them to practical industrial applications. Finally, extensive comparative experiments were conducted on the most advanced algorithms using the highly recognized penicillin simulation dataset in the industry, and the results showed the effectiveness and superiority of our proposed method.

This paper proposes a MW-Stacking AKBLS model, which provides the following main contributions:

- (1) To reduce the uncertainty of BLS in random projection, this paper innovatively introduces the approximate kernel-based BLS algorithm. By using the random fourier feature method to project feature nodes into the approximate kernel space to replace enhanced nodes, the AKBLS algorithm effectively reduces the uncertainty of random mapping, improves the nonlinear fitting ability of the model, and significantly reduces the computational cost of online applications in practical industrial process.
- (2) In order to address the challenges of the time-varying and inter batch differences in Batch Process, this paper introduces the MW-Stacking framework, which integrates multiple ABKLS models using the Stacking ensemble learning method, significantly improving the overall model's generalization ability. This framework also introduces a moving window strategy, endowing the model with adaptive ability and better adapting to slow changes in Batch Process.
- (3) This paper creatively introduces the MW-Stacking AKBLS model, which organically integrates the AKBLS algorithm with the MW-Stacking framework. This model innovatively combines the advantages of both. Through the efficient calculation and nonlinear fitting capabilities of AKBLS algorithm and the integrated learning method of MW Stacking framework, an overall solution with strong nonlinear modeling and generalization capabilities has been formed. This not only expands the applications of broad learning system in industrial, but also provides a new perspective for solving the complexity of actual production processes. And a large number of tests on the Pensim2.0 simulation platform have verified the high adaptability and excellent performance of the proposed model. The experimental results highlight the significant advantages of using the proposed soft sensing model to predict intermittent processes.

The remainder of this paper is organized as follows. “Related Work” gives the work related to the broad learning system, random fourier features and ensemble learning methods. “Proposed method” provides a thorough description of the method proposed. “Experiment” introduces the experiment and related analysis. Finally, in “Conclusions” section, we describe the conclusion.

## Related work

In this section, we will review relevant literature on BLS, random fourier features, and ensemble learning method.

### Broad learning system

In recent years, neural networks have achieved significant results in various fields, especially in complex deep neural networks^41^. However, the training of these deep networks usually requires a lot of time and is easy to getting stuck in local optima^42^. In contrast, Chen proposed an innovative model, BLS (broad learning system)^21^, based on RVFL (random vector functional link)^43^. The core concept is to transform input data through random weights and biases, first constructing feature nodes, and then connecting them to form enhanced nodes through random parameters and activation functions. Finally, the weights of the output layer are determined through fast pseudo inverse learning. Compared to complex deep neural networks, BLS performs better in computational efficiency, especially due to the use of fast pseudo inverse learning. Its unique incremental learning approach allows for rapid node expansion without the need for retraining when enhancing the network, thereby improving the flexibility of the model.

BLS has gained a lot of attention due to its excellent performance and high efficiency, and more and more variants have been developed. For example, Yang et al. integrated the prior distribution information of data into a weighted scheme to address the issue of imbalance problem, and proposed weighted WBLS (WBLS)^44^. Yu proposed a KBLS model, replacing the enhanced nodes in BLS with feature projections learned through kernel functions^35^. On this basis, Chen further proposed a double kernel-based broad learning system, in which a double kernel map-ping mechanism is utilized instead of two random mappings of BLS to generate more robust features, further improving the performance of the BLS model^45^. With its excellent problem-solving speed and concise structure, BLS has emerged as a promising alternative.

### Random fourier features

Ali Rahimi proposed a method called random fourier features^36^ to find low dimensional mappings for any given dataset, so that the dot product of the mapped data points approximates the kernel similarity between them.

Let $p(w)$ denote the Fourier transform of the kernel function $k\left( {x - y} \right)$, i.e.

1 $$ k\left( {x - y} \right) = \int {p\left( w \right){\text{exp}}\left( {jw^{{ \top }} \left( {x - y} \right)} \right)dw.} $$

According to Bochner’s theorem^46^, $p(w)$ is an effective probability density function, provided that the kernel function is continuous and positively definite.

Let $w$ be a d-dimensional vector sampled from $p(w)$. The kernel function can be approximated as

2 $$ k\left( {x,y} \right) = E_{w} \left[ {f\left( {w,x} \right)^{{ \top }} f\left( {w,y} \right)} \right], $$

where

3 $$ f\left( {w,x} \right) = \left( {\cos \left( {w^{{ \top }} x} \right),\sin \left( {w^{{ \top }} x} \right)} \right)^{{ \top }} . $$

We can approximate the expectation in (1) by dividing the empirical mean by $m$ Fourier components $w_{1} ,...,w_{m}$, samples from the distribution $p(w)$ and obtain the low dimensional representation of point $x$:

4 $$ z\left( x \right){ = }\frac{1}{\sqrt m }\left( {\cos \left( {w_{1}^{{ \top }} x} \right), \ldots ,\cos \left( {w_{m}^{{ \top }} x} \right),\sin \left( {w_{1}^{{ \top }} x} \right), \ldots ,\sin \left( {w_{m}^{{ \top }} x} \right)} \right). $$

Using this mapping, the kernel matrix $K$ can be approximated as

5 $$ \Omega = \hat{K}{ = }H^{{ \top }} H, $$

where

6 $$ H{ = }\left[ {z\left( {x_{1} } \right)^{{ \top }} , \ldots ,z\left( {x_{n} } \right)^{{ \top }} } \right]. $$

### Ensemble learning method

As an efficient algorithm, BLS can consider using ensemble learning frameworks to improve the robustness of the model. Ensemble learning, as an important branch of machine learning, achieves powerful model performance improvement by integrating multiple learning models. Research shows that the integration of multiple learners can effectively reduce the bias and variance of a single model, thus having better generalization ability in the face of unknown data^47^. Therefore, in recent years, more and more scholars have begun to combine broad learning with ensemble learning. Taking Wang’s work as an example, the bagging ensemble method was used to integrate 100 sub BLS models^48^, demonstrating the application of broad learning in the ensemble framework. In addition, Liu proposed a new adaptive incremental structure, namely stacked BLS, which proved that this integrated structure can significantly reduce the number of nodes and training time of the original BLS^49^. Overall, combining ensemble learning with broad learning is not only a trend, but also a highly promising research direction. This fusion makes the model more flexible when facing different application scenarios, enabling better selection and optimization of models, thereby achieving superior performance. In future research, we can further explore the application of different ensemble learning methods in broad learning to further improve the performance and adaptability of the model.

## Proposed method

In this section, we will provide a detailed introduction to the AKBLS algorithm and the MW-Stacking framework. Table 1 lists some symbols used in this paper.

**Table 1:** Notation in the proposed method.

Notation	Size	Description
$N$	/	The number of training samples
$M$	/	The number of feature dimensions
$n$	/	The number of the feature groups
$n_{f}$	/	The number of feature nodes in each group
$n_{F}$	/	The total number of feature nodes
$D$	$N \times M$	The training data set
$Z$	$N \times n_{f}$	Feature nodes
$\Omega$	$N \times N$	Approximate kernel matrix
$W_{ei}$	$M \times n_{f}$	Weights for the $i$-the group of feature nodes
$W_{\Omega }$	$n_{F} \times N$	The reconstruction weights for the link between feature nodes and approximate kernel matrix
$W$	$\left( {n_{F} + N} \right) \times 1$	Weights for the output layer
$w_{m}$	/	The number of moving window length
$l$	/	The number of moving step size

### Approximate kernel-based broad learning system

**Figure 1:**
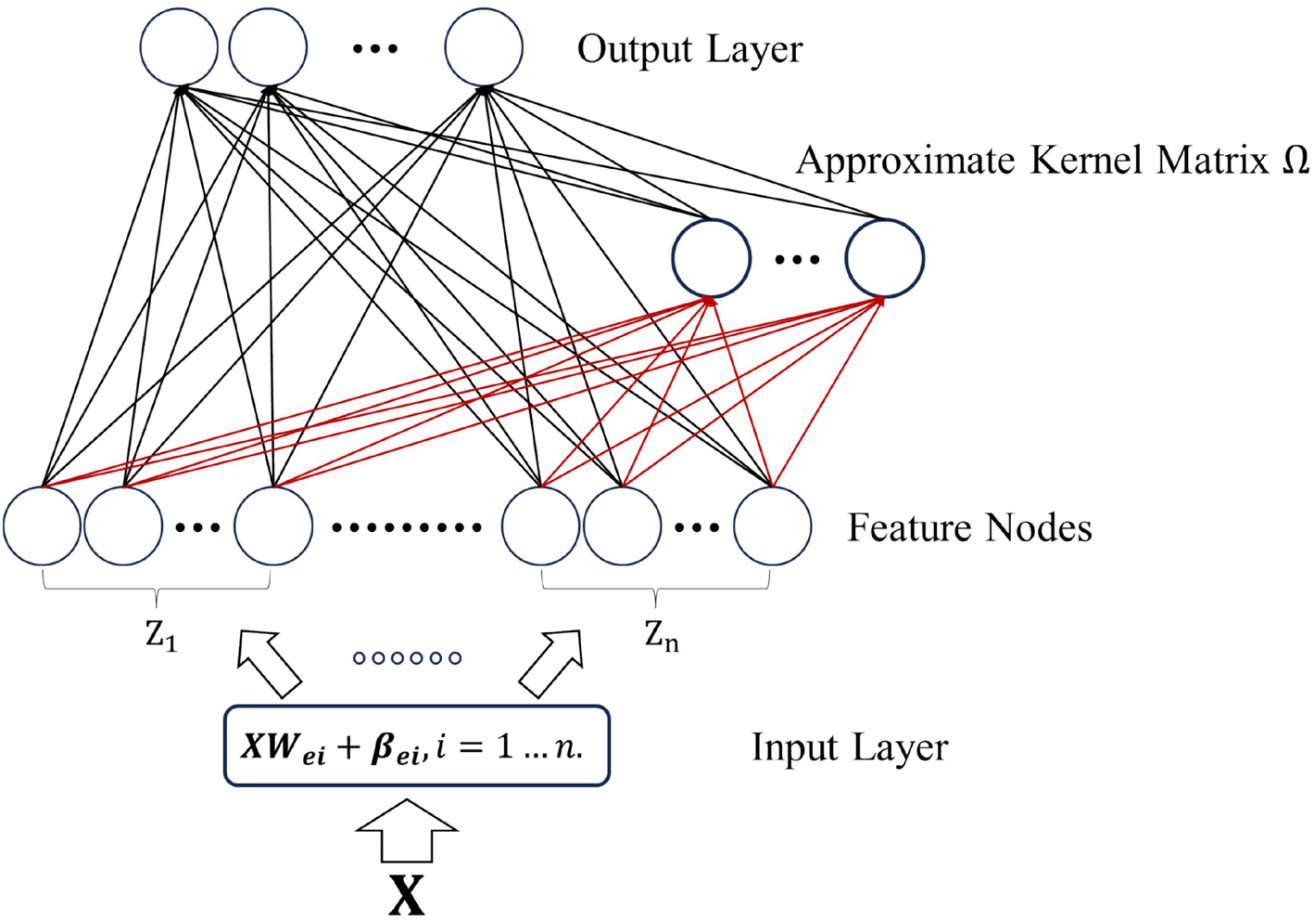
Overview framework of proposed AKBLS.

Traditional BLS generates feature nodes and enhancement nodes through two random mappings. Due to the generation of features in random mapping, redundant features and outliers may be introduced during the mapping process. This has a significant impact on the performance of the model. In response to this issue, this paper proposes the approximate kernel-based broad learning system. Figure 1 shows the overview framework of AKBLS proposed in this paper. Assuming the training dataset is ${\text{D = }}\left\{ {\left( {{\text{x}}_{i} ,y_{i} } \right)} \right\}_{i = 1}^{N}$, where $N$ is the size of the number of samples in the dataset, and the samples exist in the M-dimensional space $R^{M}$. The training process of AKBLS consists of three steps.

Firstly, $n$ sets of feature nodes are randomly generated. Assuming that we project the input $X$ with a shape of $N \times M$ into a new feature space considered as $i$ th feature node group by $W_{ei} \left( {M \times n_{f} } \right)$ and $\beta_{ei} \left( {M \times 1} \right)$. Therefore, $X$ is mapped to a random space by

7 $$ Z_{i} { = }\phi_{i} \left( {XW_{ei} { + }\beta_{ei} } \right),i \, = \, 1 \, to \, n. $$

where $\phi_{i}$ is the nonlinear activation function.

Due to the unsupervised feature construction of each random mapping, the model exhibits unpredictability. To reduce this unpredictability, it is necessary to use multiple feature mappings to verify the integrity of the input. Next, combine and connect all feature nodes to form a feature layer $Z$. Due to the fact that the feature layer is randomly generated, without label guidance or adjustment, the next constructed enhancement layer should have the ability to guide feature extraction.

In the second step, first use Eq. (4) to map the feature layer $Z$ to the low dimensional space $H$, and then put the obtained $H$ into Eq. (5) for calculation to obtain the approximate kernel matrix $\Omega$. Next, by replacing the enhanced nodes with the calculated approximate kernel matrix $\Omega$, where the approximate kernel function is a Gaussian kernel. Usually, data in the original space may not have linear separability. By using the method of approximating the kernel matrix, we can map the data to a high-dimensional feature space, significantly reducing the redundant features and outliers that may be introduced during the mapping process from feature nodes to enhancement nodes, and this does not significantly increase the computational complexity.

$\Omega$ can be evaluated using kernels as shown in Eq. (5) when the conditions for Mercer’s theorem are met. In this study, we focus on using random fourier feature methods to approximate radial basis function (RBF) kernels.

8 $$ K\left( {H_{k} ,H_{m} ,\sigma } \right) = \exp \left( { - \frac{{\left\| {H_{k} - H_{m} } \right\|^{2} }}{{2\sigma^{2} }}} \right). $$

Once the kernel matrix is set, the next step is to calculate the transformation matrix from feature nodes to the kernel matrix. The approximate kernel BLS autoencoder (AKBLS-AE) is responsible for learning the transformation from kernel matrices to feature nodes. The output layer and input layer of AKBLS-AE are both feature nodes. Unlike traditional algorithms, AKBLS-AE is more flexible in minimizing both reconstruction error and weight norm simultaneously. The calculation of weight solutions can be considered as a problem for optimizing the following loss functions^50^:

9 $$ \mathop {\arg \min }\limits_{{W_{\Omega } }} { : }\left\| {ZW_{\Omega } - \Omega } \right\|_{2}^{2} + \lambda_{1} \left\| {W_{\Omega } } \right\|_{2} $$

where, $W_{\Omega }$ is the weight connecting the feature nodes $Z$ to the approximate kernel matrix $\Omega$, and $\lambda_{1}$ is the tradeoff parameter. In AKBLS-AE, pseudo inverse can be used as an effective method for solving weights, and its solution can be approximately equal to ridge regression^51^. According to ridge regression, add a positive value $\left( {1/C} \right)$ on the diagonal of $Z^{{ \top }} Z$ to regularize the value of $W_{\Omega }$. Therefore, in order to calculate the weight $W_{\Omega }$, we have

10 $$ W_{\Omega } = Z^{{ \top }} \left( {CI + ZZ^{{ \top }} } \right)^{ - 1} \Omega . $$

In the third step, in order to enrich the features, the feature nodes and enhancement nodes (the approximate kernel matrix) are merged as hidden layers by connecting them:

11 $$ A{ = }\left[ {Z,\Omega } \right] $$

The weight of the output layer is represented as $W$. Assuming the given label is $Y\left( {N \times 1} \right)$, the following optimization problem can be considered to find the solution to $W$:

12 $$ \mathop {\arg \min }\limits_{W} { : }\left\| {AW - Y} \right\|_{2}^{2} + \lambda_{2} \left\| W \right\|_{2}^{2} . $$

where, $\lambda_{2}$ represents the constraint on the sum of squared weights. Ridge regression theory is used to solve this problem. Therefore, we have

13 $$ W{ = }A^{ + } Y $$

where, $^{ + }$ represents the pseudo inverse operator. The pseudo inverse of matrix A is defined as

14 $$ A^{ + } { = }\lim_{\lambda \to 0} \left( {\gamma I + AA^{{ \top }} } \right)^{ - 1} A^{{ \top }} $$

According to Eqs. (12) and (13), obtain the output weight $W$, and the training process of AKBLS is completed. When the predicted label is represented as $Y^{*}$, the result is obtained from the following equation:

15 $$ Y^{*} = AW $$

### Stacking AKBLS

In the previous text, AKBLS was designed to express the nonlinear features of the original space while reducing the uncertainty caused by random mapping in the original BLS. In industrial production, the complexity and constantly changing characteristics of data require models to have stronger generalization ability. In response to this requirement and to further improve the performance of the model, this paper introduces the Stacking ensemble framework. For the same prediction task, different learners have certain differences in performance, and Stacking ensemble learning can comprehensively utilize the advantages of multiple learners to approximate the optimal solution, making the model more robust to adapt to different industrial scenarios. This ensemble method integrates the prediction results of multiple primary learners and trains a meta learner to use these prediction results to produce more accurate predictions.

**Figure 2:**
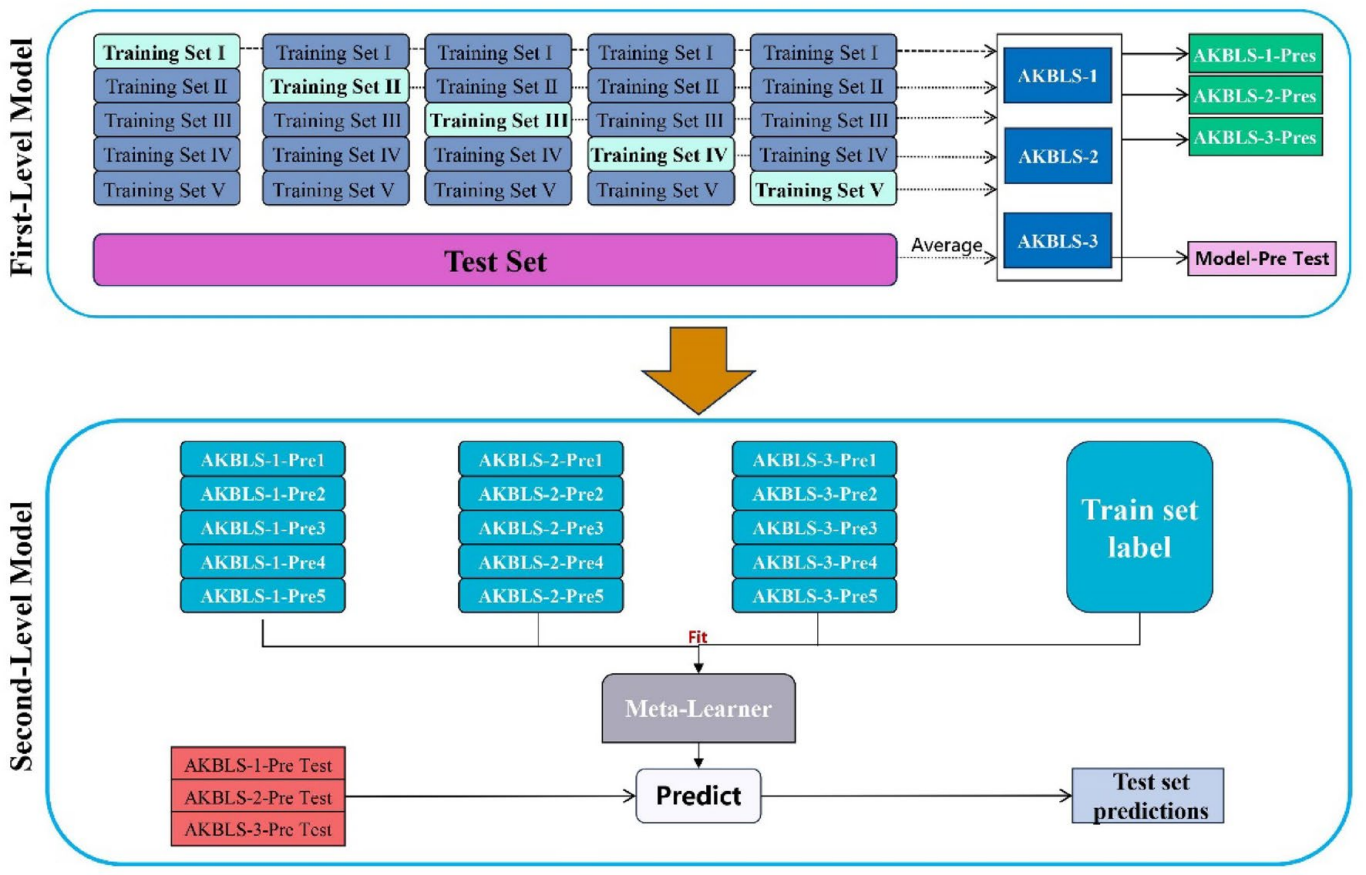
The stacking AKBLS model framework used in this study.

Stacking ensemble learning method is a machine learning technique based on model combination, used to improve the performance of prediction models. It trains a meta learner to integrate the predicted results of multiple primary learners as input, resulting in more accurate predictions. The primary learner can use different algorithms or have different hyperparameter settings, while the meta learner can be any machine learning model, such as logistic regression, decision tree, or neural network. This choice is based on the simplicity, interpretability, and computational efficiency of linear regression models, while also providing good performance in many cases, helping to improve computational efficiency and reduce the risk of model overfitting. Stacking achieves better performance in complex prediction tasks by combining the predictive abilities of multiple models. Cross validation methods are used to reduce the risk of overfitting during model training. The principle of the Stacking based integration framework in this paper is shown in Fig. 2.

### MW-stacking AKBLS

In fact, many existing industrial batch process often have time-varying production processes, and the working conditions will change over time, such as equipment aging, process drift, etc., which can lead to changes in the entire production process and a decrease in the accuracy of previous prediction models. In the face of the urgent need for model adaptability in industrial batch process, this paper further develops the stacking AKBLS model previously proposed. We have designed an adaptive framework based on the moving window method to address the characteristics of data variability, nonlinearity, and inter batch differences, and named it the MW-Stacking framework. This model not only inherits the advantages of Stacking ensemble learning, improves the model performance by integrating the prediction results of multiple primary learners, but also makes further innovation in terms of adaptability. Based on the moving window mechanism, new data is continuously added to the training samples through the moving data window, while also removing a corresponding amount of sample data used for modeling the original model, forming a new training dataset. Retrain the original model using the newly collected dataset. Through this approach, the new model established is more in line with the current operating conditions of the system, and the detection results for abnormal situations are more accurate. This can timely capture the changing trends in industrial Batch Process and maintain the model's adaptability to time-varying and batch differences. The introduction of this adaptive framework provides an effective solution for the model to better cope with complex and ever-changing production environments in practical industrial applications.

If the length of the moving window is $w_{m}$ and the moving step is $l$, the sample data in the $m$th moving window is:

16 $$ D_{m} = \left\{ {\left( {x_{ml} ,y_{ml} } \right),...,\left( {x_{{ml + w_{m} }} ,y_{{ml + w_{m} }} } \right)} \right\} $$

**Figure 3:**
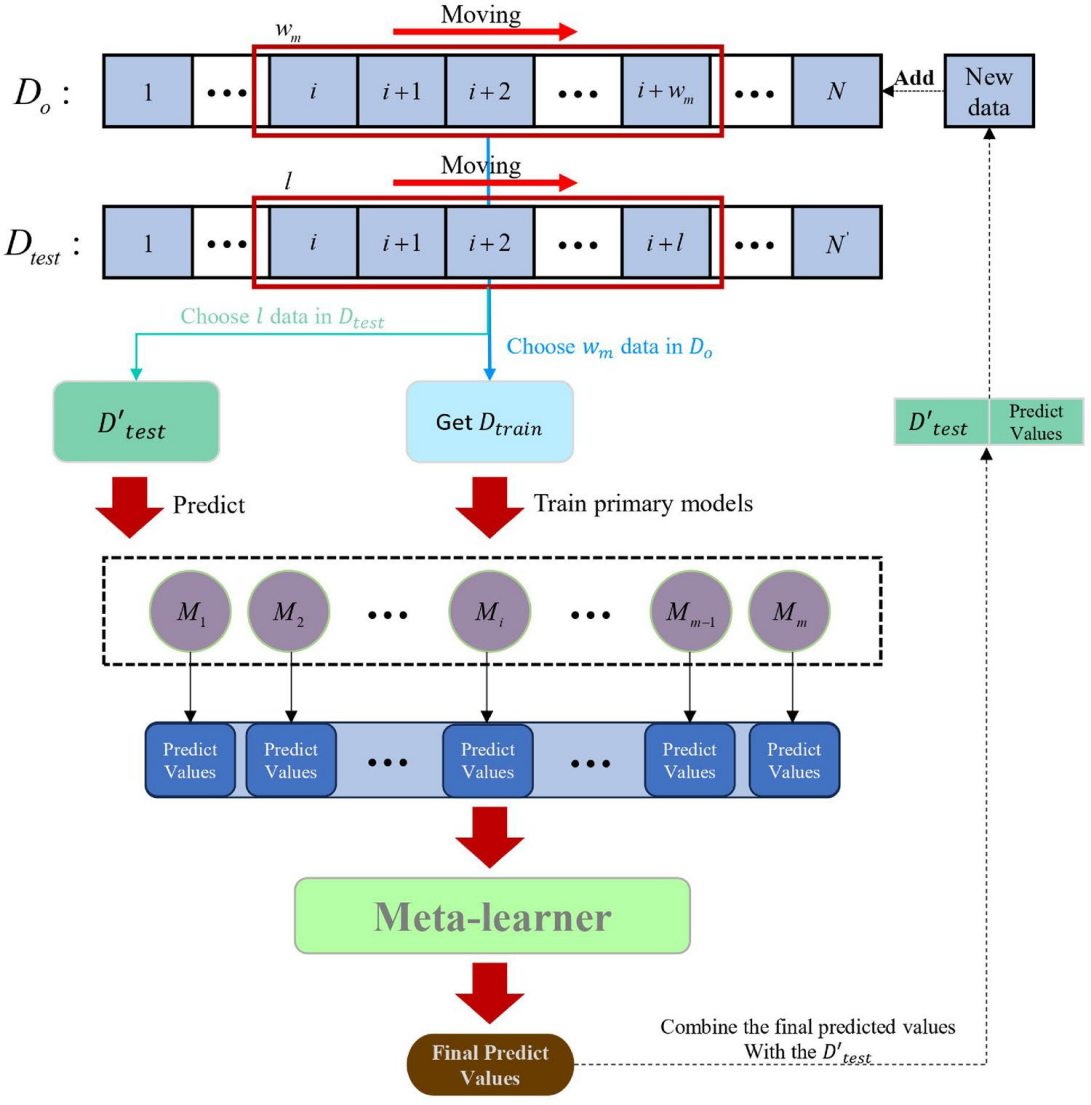
Model flowchart of MW-stacking AKBLS model.

We start by initializing the dataset and parameters, following which a moving window selects $w_{m}$ instances from the original training database to establish the initial training dataset. We train a triad of approximate kernel-based broad learning system (AKBLS) on this newly established training dataset, utilizing them as primary learners to construct a Stacking ensemble model that enhances the model's generalization capabilities. Utilizing a step size $l$, we select $l$ instances from the original test database to establish the initial test dataset. The constructed Stacking-AKBLS model is then applied to predict this test set, and its latest prediction outcomes are integrated back into the original training database to enrich it. Then, move the window to move the $l$ steps towards the latest data in the original training database, select a new set of $w_{m}$ instances to establish the training dataset for the next iteration, and start the next cycle. The detailed flowchart is shown in Fig. 3.

As the moving window continues to move forward, the system establishes new Stacking AKBLS models in different windows to dynamically update the models and better reflect the real-time operation of the system. Algorithm 1 provides a detailed explanation of the training process for MW-Stacking AKBLS.

**Algorithm 1:**
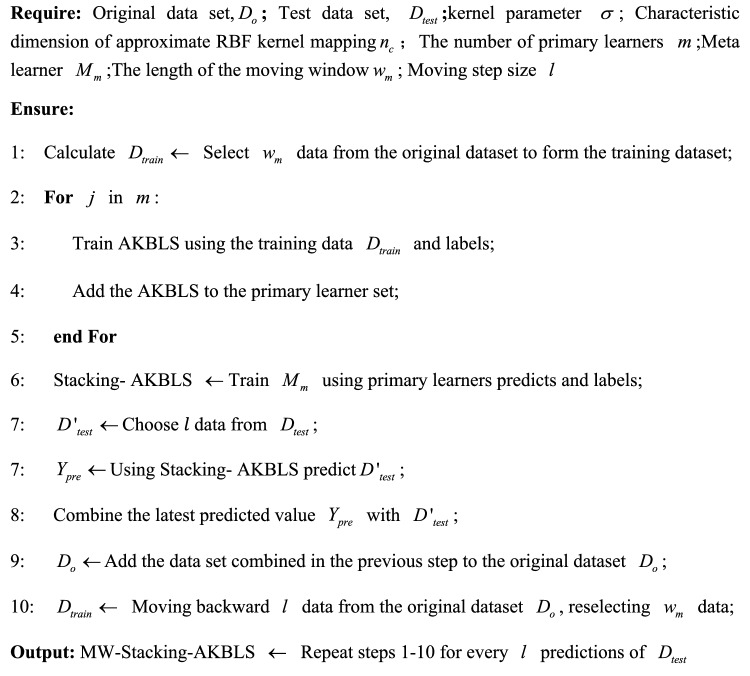
MW-Stacking-AKBLS

## Experiment

In this section, this paper will conduct a comprehensive performance comparison between the proposed AKBLS and Yu’s KBLS methods, as well as traditional BLS methods. This paper aims to comprehensively evaluate the performance differences of these methods through extensive testing on multiple datasets of varying scales. Furthermore, this paper will separately integrate the MW-Stacking framework with AKBLS, KBLS, and BLS methods, and compare their effectiveness in detail to fully demonstrate the excellence of the MW-Stacking AKBLS model. In addition, we will compare the MW-Stacking AKBLS model with other commonly used machine learning models, deep learning, and a series of adaptive models in the industrial field to comprehensively evaluate the effectiveness of the proposed method in this paper. This series of comparisons will provide a profound understanding of the performance and applicable scenarios of different methods in various aspects.

### Data source

In the comparison and evaluation section of the AKBLS method, we selected multiple datasets of varying sizes, all of which were sourced from the Kaggle website and simulation platform. Through this series of experiments, we aim to comprehensively evaluate the performance differences between the proposed method and KBLS and BLS methods. The specific dataset information used has been summarized in Table 2, where N denotes the number of samples in the dataset, M denotes the number of attributes.

**Table 2:** Experimental dataset details.

Name of data set	N	M	Abbreviation
Penicillin dataset 1	800	15	Penicillin dataset 1
Penicillin dataset 2	1600	15	Penicillin dataset 2
Penicillin dataset 3	5200	15	Penicillin dataset 3
Penicillin dataset 4	10000	15	Penicillin dataset 4
Steel industry energy consumption	10945	19	Steel industry
Flight price prediction	14032	39	Flight price
Electric power consumption	11377	27	Electric consumption
Remaining useful lifetime prediction	15064	8	Remaining useful life

The penicillin fermentation data used in this study source the Pensim2.0 simulation platform (http://simulator.iit.edu/web/software.html), which was specifically designed to simulate the penicillin fermentation process^52^. This software supports simulating penicillin fermentation by setting parameters such as substrate concentration, CO_2_ concentration, PH value, fermenter temperature, and medium volume. In this experiment, with product concentration as the main variable, this study selected 15 input parameters: air flow, agitator power, flow rate, acid intake, alkali intake, cooling water flow, hot water flow, substrate concentration, oxygen concentration, biomass concentration, medium volume, CO_2_ concentration, PH, fermenter temperature, and heat production. The sampling interval is set to 1 h. Collect a dataset of penicillin fermentation process by simulating various conditions during the fermentation process.

The computer configuration used for soft sensing modeling is as follows: Windows 10 Ultimate (64 bit); CPU: Intel (R) Core (TM) i7-4790 (3.60 GHz, 3.60 GHz); RAM: 12.0 GB; PyCharm version: 2022.

### Comparison and evaluation of AKBLS algorithm performance

In order to evaluate the performance of the proposed AKBLS algorithm in practical applications, extensive testing was conducted on multiple publicly available datasets. These datasets are mostly selected from datasets related to industrial production process or datasets with high dimensional and nonlinear characteristics similar to batch process. On the training set, a five-fold cross validation method was used to determine the optimal parameter configuration for all algorithms. Subsequently, once the optimal parameters are determined, they are fixed and tested on an independent test set. In order to evaluate the performance of the three algorithms, the $R^{2}$ was introduced, which is widely used to evaluate regression prediction algorithms. The calculation formula is as follows:

17 $$ R^{2} = 1 - \frac{{\sum\nolimits_{i = 1}^{{N_{test} }} {\left( {\hat{y}_{i} - y_{i} } \right)}^{2} }}{{\sum\nolimits_{i = 1}^{{N_{test} }} {\left( {y_{i} - \overline{y}_{i} } \right)^{2} } }} $$

Among them, $y_{i}$ is the actual output, $\hat{y}_{i}$ is the estimated output, $\overline{y}_{i}$ is the average of the actual output, and $N_{test}$ represents the number of test samples.

In this study, $R^{2}$ can be used to quantify the performance of the AKBLS algorithm compared to the other two algorithms (KBLS and BLS). This helps to provide an intuitive and objective comparison, allowing readers to have a clearer understanding of AKBLS’s predictive ability on different datasets, to comprehensively and accurately evaluate the superiority of the proposed method, and to provide strong support for further research and application. The performance of all algorithms is evaluated by the average accuracy of ten runs of testing. Meanwhile, considering the crucial importance of model timeliness, this article also calculated the training time of each algorithm on various datasets. This comprehensive evaluation aims to provide readers with comprehensive information, enabling them to fully understand the performance of the AKBLS algorithm in practical application scenarios.

**Figure 4:**
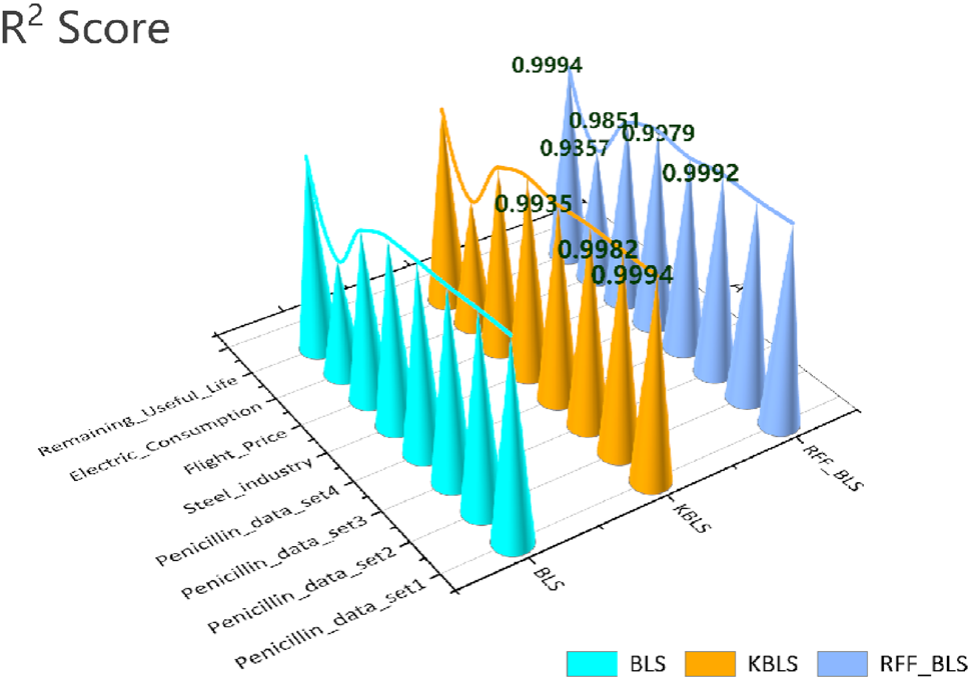
R^2^ scores of AKBLS, KBLS and BLS in different datasets.

**Figure 5:**
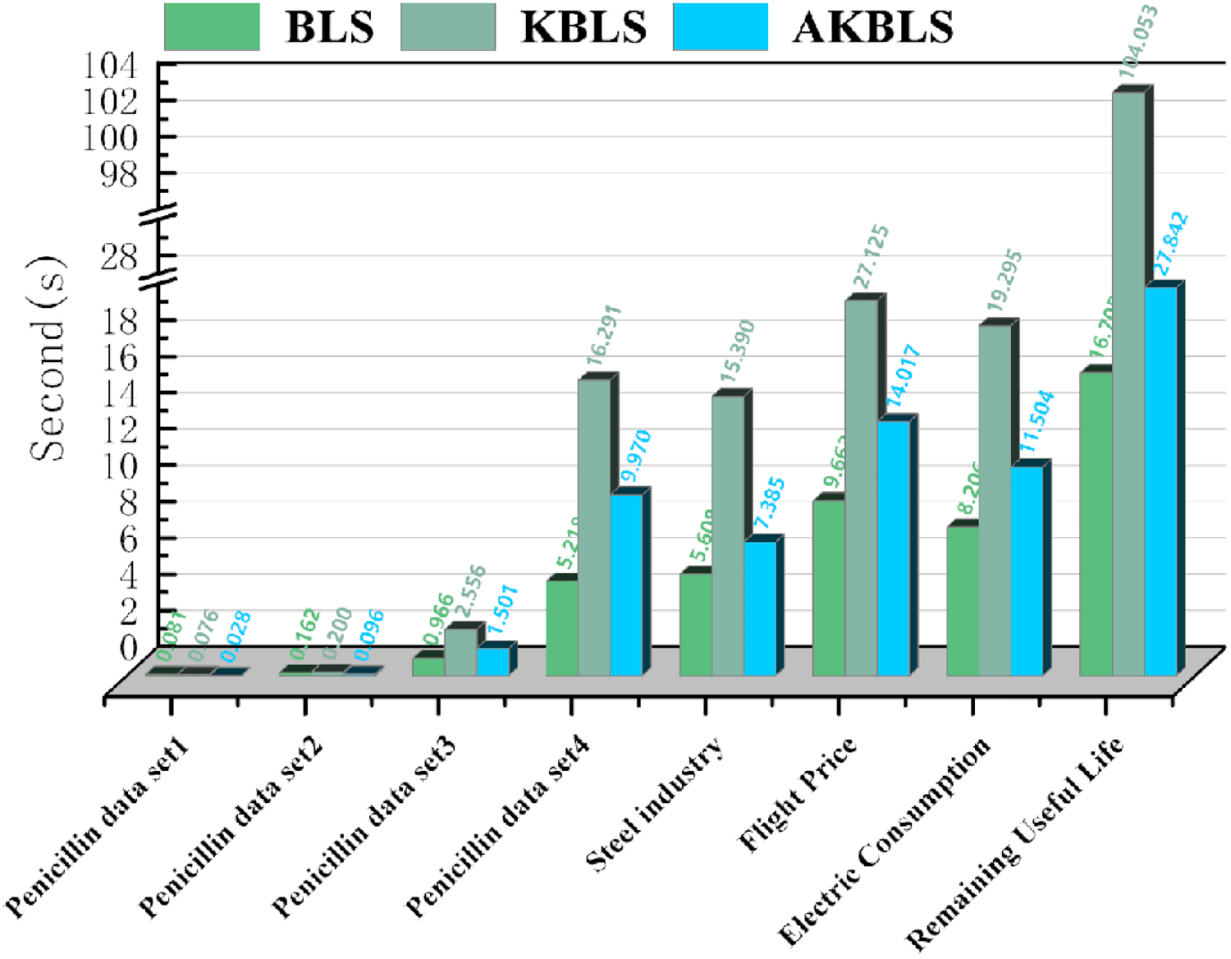
Training time of AKBLS, KBLS and BLS in different datasets.

As shown in Fig. 4, with the expansion of the dataset size, traditional BLS algorithms are gradually at a disadvantage, while the AKBLS algorithm proposed in this paper has slightly higher scores than the KBLS algorithm proposed by Yu, and both are significantly better than traditional BLS algorithms. In Fig. 5, this article compares the training time of three methods on different publicly available datasets. It can be seen that as the data size expands, the AKBLS algorithm proposed in this article has a significant decrease in training time compared to KBLS, with a maximum training time reduced by 75%. Table 3 presents in detail the scores and training times of the three algorithms on different publicly available datasets. It can be seen that the prediction accuracy of the AKBLS algorithm proposed in this paper is slightly higher than that of KBLS and significantly higher than that of BLS. In the study of Yu et al., the reason for this phenomenon is given^35^. The reason is that BLS enhancement nodes are derived from a random mapping of feature nodes, resulting in linear combinations that may introduce redundancy. In contrast, KBLS computes enhancement nodes using kernel projections, which create new features that measure similarity among samples. As a result, KBLS enhancement nodes contain richer information. The training time is slightly higher than that of BLS due to the calculation of kernel matrix. But, compared with KBLS, the training time can be significantly reduced, which effectively proves the performance superiority of the AKBLS method proposed in this paper.

**Table 3:** Performance of different algorithms on real-world data sets.

Name of data set	BLS		KBLS		AKBLS	
	${\text{R}}^{{2}}$	Time	${\text{R}}^{{2}}$	Time	${\text{R}}^{{2}}$	Time
Penicillin dataset 1	0.99774	0.08096	0.99938	0.07596	0.99924	0.02797
Penicillin dataset 2	0.99538	0.16213	0.99823	0.19988	0.99782	0.09594
Penicillin dataset 3	0.99132	0.96596	0.99742	2.55625	0.99918	1.50113
Penicillin dataset 4	0.98983	5.21843	0.99355	16.2907	0.9934	9.97005
Steel industry energy consumption	0.98718	5.60765	0.99788	15.3896	0.99793	7.38505
Flight price prediction	0.97042	9.66289	0.98484	27.125	0.98512	14.0166
Electric power consumption	0.91691	8.2057	0.9288	19.2953	0.9357	11.5036
Remaining useful lifetime prediction	0.99645	16.7048	0.9994	104.052	0.99944	27.8417

### Effect of MW-stacking framework

A study suggests that as the number of primary learners increases, the performance of stacked ensemble models improves^53^. When the number of primary learners reaches four, the Stacking ensemble model exhibits a higher R^2^ score. However, compared to the Stacking ensemble model with three primary learners, the improvement in R^2^ score is minimal. In addition, the computational burden generated by the accuracy and diversity calculations within the aggregation model leads to a stable and predictable increase in computation time. Based on this, this paper has conclusively selected three primary learners to construct the ensemble model. The main purpose of this choice is to enhance model performance, concurrently minimizing computation time and averting overfitting concerns. As for the meta-learner within the ensemble model, we have chosen to employ a gaussian process regression (GPR) model. This decision is motivated by the superior capabilities of GPR in capturing complex patterns, providing uncertainty estimates, and adapting well to various data distributions. The inherent flexibility and non-linearity of GPR make it well-suited for diverse modeling scenarios. Additionally, GPR models have demonstrated commendable performance across different cases, contributing to heightened computational efficiency and a diminished risk of model overfitting in comparison to linear regression models.

In order to demonstrate the effectiveness of the MW-Stacking framework, this paper used the Pensim2.0 simulation platform to collect 20 batches of penicillin fermentation datasets with different initial conditions for training the model, and collected 1 batch of datasets as the test set. To evaluate the performance of the model, this paper selects two indicators, $R^{2}$ and $RMSE$. Root Mean Square Error ($RMSE$) is a statistical measure used to measure the difference between predicted values and actual observations. In regression analysis, $RMSE$ is often used to evaluate the predictive performance of a model. The general step in calculating $RMSE$ is to square the prediction error of the model for each observation, then take the average value, and finally take the square root of the result. The calculation formula is as follows:

18 $$ RMSE = \sqrt {\frac{1}{{N_{test} }}\sum\nolimits_{i = 1}^{{N_{test} }} {\left( {\hat{y}_{i} - y_{i} } \right)^{2} } } $$

Among them, $N_{test}$ is the number of samples, $y_{i}$ is the actual output, and $\hat{y}_{i}$ is the estimated output. Therefore, the smaller the value of $RMSE$, the smaller the difference between the predicted and actual observed values of the model, and therefore the better the performance of the model.

**Figure 6:**
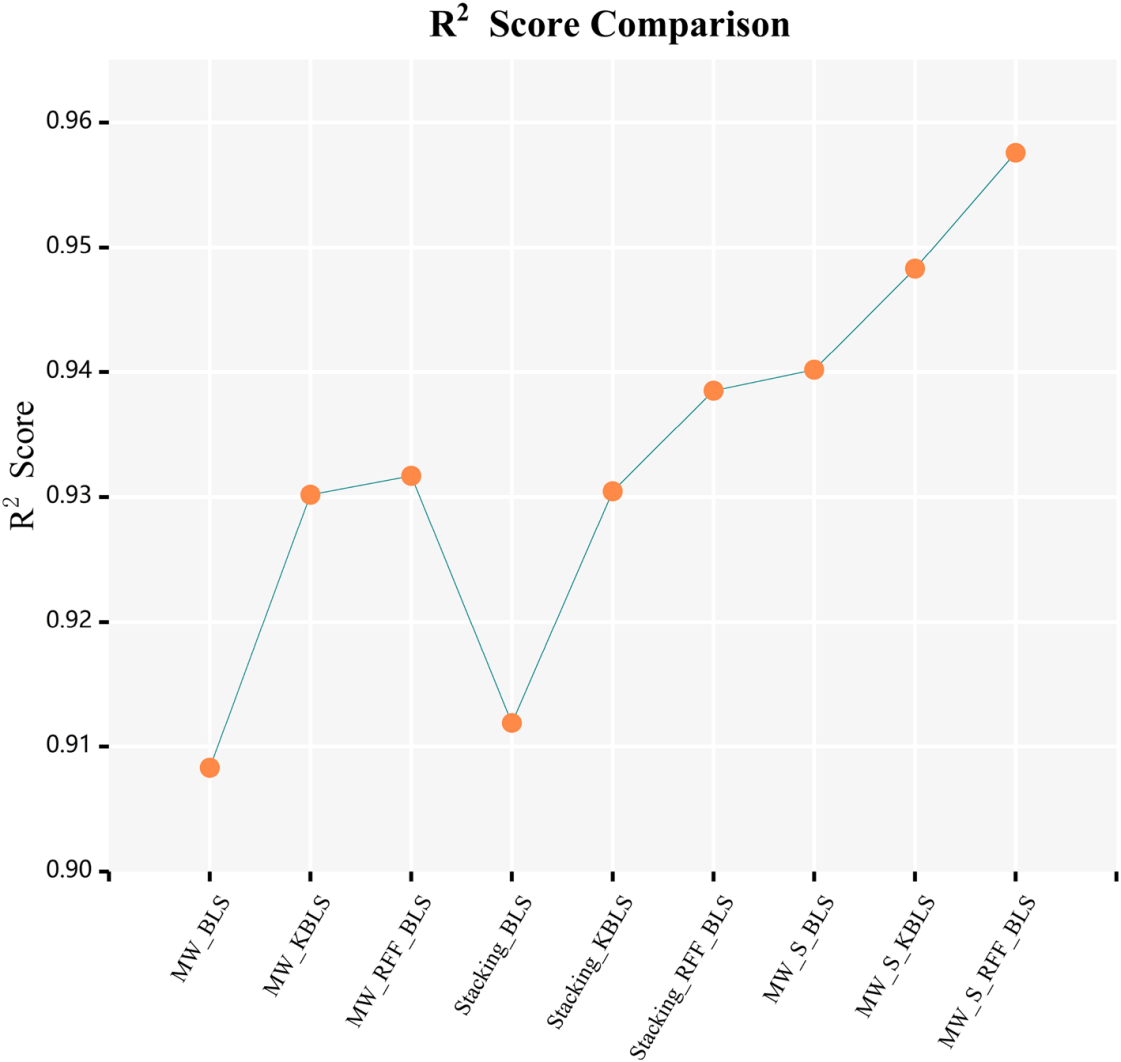
R^2^ scores of BLS, KBLS and AKBLS under different frameworks.

**Figure 7:**
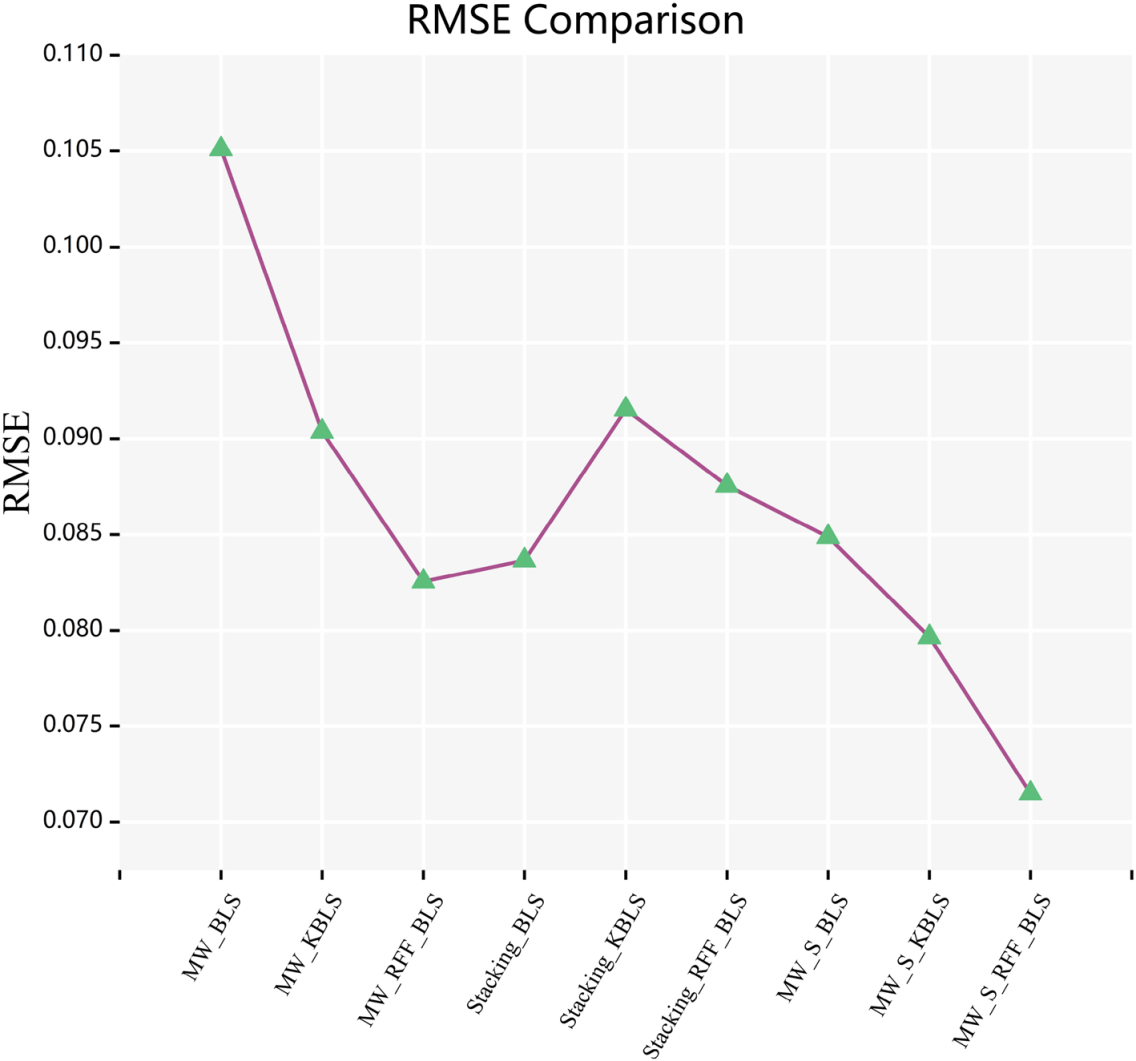
RMSE scores of BLS, KBLS and AKBLS under different frameworks.

In Figs. 6 and 7, the curves of $R^{2}$ score and $RMSE$ calculated by combining various frameworks with traditional BLS algorithm, Yu’s KBLS algorithm, and the AKBLS algorithm proposed in this paper on the penicillin fermentation dataset are shown. Firstly, only the moving window method will be combined with BLS, KBLS, and the proposed AKBLS algorithm to enhance the adaptive ability of each model. Then, we will only combine Stacking ensemble learning with these three algorithms separately. Finally, the MW-Stacking framework proposed in this article will be combined with three algorithms to calculate their respective $R^{2}$ scores and $RMSE$. The results show that regardless of the combination with any algorithm, the MW-Stacking framework proposed in this paper can significantly improve the predictive performance of the model and reduce fitting errors. Within this framework, the MW-Stacking-AKBLS model, which integrates with the previously proposed AKBLS algorithm in this paper, achieved the highest $R^{2}$ score and the lowest $RMSE$. In comparison to the second-best performing MW-Stacking-KBLS model, not only did the $R^{2}$ score improve, but $RMSE$ also decreased by 10.2%. Therefore, combining the MW-Stacking framework with the AKBLS algorithm proposed in this paper maximizes the advantages of the framework, leading to the overall optimization of predictive model performance.

### Comparison with other algorithm

In this section, we conducted a comprehensive evaluation of the proposed MW-Stacking AKBLS model, aiming to verify its effectiveness in practical applications. Using the Pensim2.0 simulation platform, we collected a dataset consisting of 20 batches of penicillin production fermentation as the training set and 5 batches as the testing set. To better fit the actual production situation of industrial batch process, we applied some noise treatment to the training dataset. At the same time, significant changes were made to the initial state of a batch of test set data, such as substrate concentration and reactor volume, compared to the first four batches of data, to test the generalization performance of the proposed model.

In order to comprehensively examine the adaptability of the proposed model to the time-varying nature of industrial batch process, we made regular changes to the initial state of the data in the test dataset. This design aims to evaluate the performance of the MW-Stacking AKBLS model in the face of complex and time-varying situations in industrial batch process.

In the industrial field, we compared common machine learning models such as gaussian process regression (GPR) and support vector regression (SVR), as well as deep learning models such as Multi-Layer Perceptron (MLP) and Long short-term memory (LSTM). In addition, a series of commonly used adaptive models such as JIT-PLS, JIT-GPR, JIT-KPLS also participated in comprehensive comparative testing.

**Figure 8:**
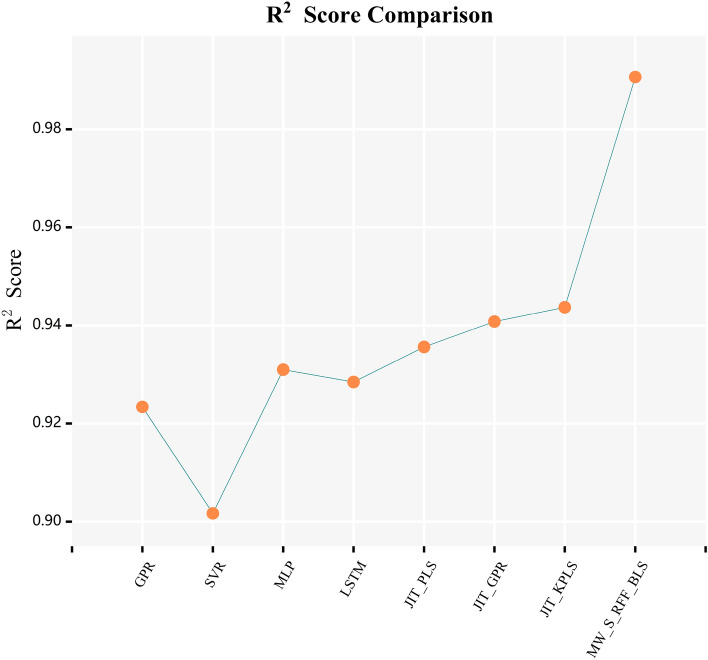
R^2^ scores of different models on a batch of test data.

**Figure 9:**
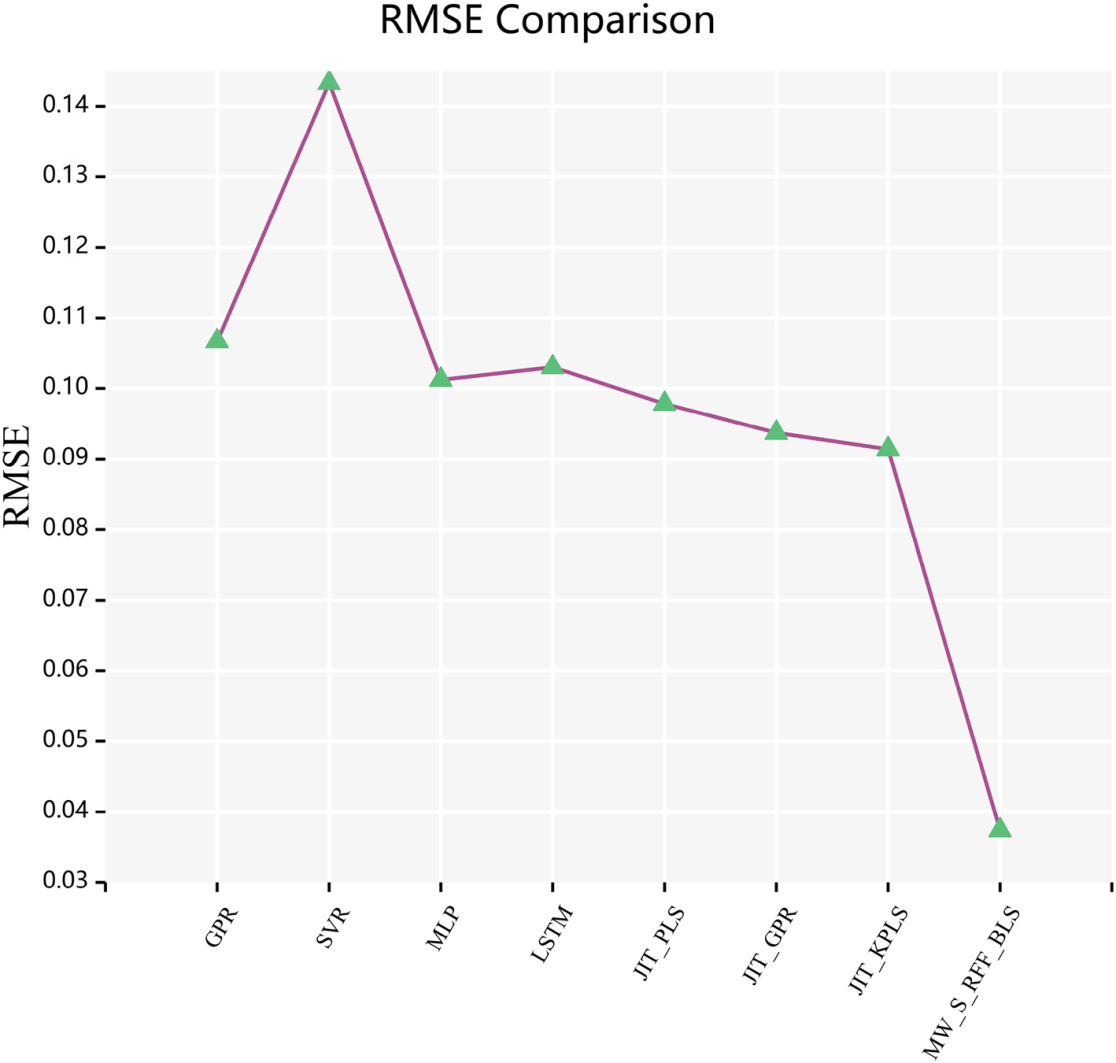
RMSE scores of different models on a batch of test data.

In Fig. 8, this article presents the $R^{2}$ scores of these 8 models on a batch of penicillin fermentation data in the test set, while Fig. 9 presents the corresponding root mean square error (RMSE). By comparing these performance indicators, we can comprehensively see the performance of each model. Obviously, the MW-Stacking AKBLS model demonstrated significant advantages, achieving the highest $R^{2}$ score (0.9906) and the lowest $RMSE$ (0.0374). In comparison to the second-best performing JIT-KPLS model with a $R^{2}$ score of 0.9437, the MW-Stacking-AKBLS model has shown a significant improvement. This fully demonstrates that the MW-Stacking AKBLS model proposed in this article can better fit data, capture changes in target variables, and achieve more accurate prediction results. The comparison results on other batches of test datasets are detailed in Tables 4 and 5. It can be seen that the MW-Stacking AKBLS model exhibits extremely high predictive performance on various types of penicillin test sets. Meanwhile, on the fifth batch of test data with significantly different initial states from other datasets, the model proposed in this paper still maintains excellent performance advantages, proving its excellent generalization performance.

**Table 4:** R^2^ scores for different models on different batch test data.

Models	Testdata1	Testdata2	Testdata3	Testdata4	Testdata5	Average value
GPR	0.9259	0.9234	0.8801	0.9144	0.7482	0.8784
SVR	0.92	0.8617	0.8341	0.9004	0.8096	0.8652
MLP	0.9337	0.931	0.8921	0.9315	0.7353	0.8847
LSTM	0.9414	0.9285	0.8496	0.8979	0.7264	0.8688
JIT_PLS	0.9074	0.9356	0.9571	0.9431	0.8261	0.9139
JIT_GPR	0.9029	0.9408	0.9614	0.9347	0.8383	0.9156
JIT_KPLS	0.9065	0.9437	0.9552	0.9485	0.8519	0.9212
Proposed model	0.9822	0.9906	0.9656	0.9488	0.8862	0.9547

**Table 5:** RMSE for different models on different batch test data.

Models	Testdata1	Testdata2	Testdata3	Testdata4	Testdata5	Average value
GPR	0.1173	0.1067	0.1209	0.1149	0.1577	0.1235
SVR	0.1219	0.1433	0.1422	0.1239	0.1371	0.1337
MLP	0.111	0.1012	0.1147	0.1019	0.1617	0.1181
LSTM	0.1043	0.103	0.1354	0.1244	0.192	0.1318
JIT_PLS	0.1311	0.0978	0.0723	0.0929	0.131	0.105
JIT_GPR	0.1343	0.0937	0.0686	0.1003	0.1264	0.1047
JIT_KPLS	0.1318	0.0914	0.0739	0.0883	0.1209	0.1013
Proposed model	0.0574	0.0374	0.0648	0.0888	0.1142	0.0725

Through this series of experiments, the exceptional performance of the proposed MW-Stacking-AKBLS model is clearly demonstrated in various aspects. This model not only performs well in predictive and generalization performance, but also shows significant superiority in dealing with the variability of Batch Process. Its outstanding ability to address the complex challenges of highly nonlinear dynamics and substantial inter-batch variations in industrial batch process is evident. These experimental results provide strong support for the feasibility and effectiveness of the proposed method in practical applications.

## Conclusion

Batch processes are crucial for the production and innovation of key industries in the national economy, such as fine chemicals, pharmaceuticals, biotechnology, and high-performance materials. They contribute significantly to the technological advancement and overall economic growth of other industries. However, due to the high nonlinearity and time-varying nature of production, it is difficult to ensure the consistency of product quality. Thus, this study proposes a model called MW-Stacking AKBLS to address the highly nonlinear and time-varying characteristics in Batch Process. This model innovatively proposed the AKBLS algorithm and the MW-Stacking framework. Broad learning system (BLS) emphasizes the width of the model rather than depth compared to deep learning, and achieves powerful nonlinear modeling capabilities by introducing a large number of random mapping nodes. Compared with deep learning, broad learning has less training time when processing large-scale data, making it gradually gaining attention and application in industrial processes. On the basis of the traditional BLS algorithm, Yu proposed the KBLS algorithm, which projects the feature nodes obtained from the first random mapping into the kernel space to reduce the uncertainty of random projection, improve the nonlinear fitting ability of the model, and thus improve the performance of the model. However, due to the high computational cost of kernel matrix, its online application in practical industrial processes is limited. In response to this issue, this study innovatively proposes the AKBLS algorithm, which uses the method of Random Fourier Features to approximate the kernel matrix, rather than directly calculating the kernel matrix. This not only maintains strong nonlinear fitting ability and reduces uncertainty, but also significantly reduces model training time, enhancing the timeliness of model building. And comparative experiments were conducted between BLS, KBLS, and the proposed AKBLS algorithm on a large number of publicly available datasets of different sizes, verifying the superiority of the AKBLS algorithm’s performance.

In this paper, the proposal of the MW-Stacking framework aims to address the challenges of time-varying and inter batch differences in Batch Process. By adopting the Stacking ensemble learning method, multiple ABKLS models are integrated together, significantly improving the overall model’s generalization ability. Time variability is a common problem in actual batch process, as changes in the production process are caused by equipment aging, process drift, and other factors, which affect the accuracy of previous prediction models. The introduction of moving window strategy endows the model with adaptive ability, enabling it to better adapt to slow changes in batch process. The MW-Stacking framework, through the excellent performance of ensemble learning, comprehensively adapts to the time-varying and inter batch differences of batch process, providing innovative solutions for industrial data modeling and new methods and perspectives for production processes in practical applications.

Finally, by comparing and validating with various commonly used algorithms on a large number of penicillin simulation datasets, the experimental results clearly demonstrate the significant improvement of the MW-Stacking-AKBLS model in prediction accuracy compared to other algorithms. This further confirms the effectiveness of the model in addressing the challenges in Batch Process.

Future research directions include conducting a more in-depth evaluation of the impact of random mapping on model performance in BLS and conducting theoretical analysis. In response to the urgent demand for real-time performance in industrial processes, research is being conducted on how to further enhance the online learning and real-time prediction capabilities of the MW-Stacking AKBLS model. In addition, research on reducing the computational complexity and number of parameters of BLS models will become a noteworthy topic. Last but not least, we plan to explore the trade-off between breadth and depth in BLS in the future, and effectively combine their impact on model performance. This series of studies will provide deeper theoretical support and practical guidance for the intelligence and optimization of industrial processes.

## Data Availability

The data that support the findings of this study are available from the corresponding author upon reasonable request.
